# High Fat High Calories Diet (HFD) Increase Gut Susceptibility to Carcinogens by Altering the Gut Microbial Community

**DOI:** 10.7150/jca.43561

**Published:** 2020-04-07

**Authors:** Heiying Jin, Chunxia Zhang

**Affiliations:** Department of colorectal surgery, The Second Affiliated Hospital of Nanjing University of Chinese Medicine, 23Nanhu Road, Nanjing 210017, China

**Keywords:** microbial community, colorectal cancer, susceptibility, prevention

## Abstract

**Objective**: To investigate the risk of colorectal cancer and its relationship with colonic flora and microenvironment under high-fat and high-calorie diet. Methods: Wistar rats were used to study, and they were given normal diet, high-fat diet, and dimethyl hydrazine (DMH) to induce the occurrence of colorectal cancer. Then observe the difference in tumor formation and the relationship among microbial community, inflammatory factors and metabolism. Results: No tumors were found in the normal diet group (G1) and the high-fat diet group (G3). Four nodules were found in the four rats in the normal diet + DMH group (G2) and 8 cancerous nodules were formed in 7 rats (70%) from high-fat diet + DMH group (G4). Cholesterol and TNF-α increased, IL-1, IL-6 and LEP decreased in the high-fat diet group. The difference was statistically significant. In the cancer-inducing group, only the difference in cholesterol was statistically significant. Compared with the normal diet group (G1) and the high-fat diet group (G3), the rat's gut bacterial abundance was not significantly different, but the gut flora structure was significantly changed. The content of Candida in the intestinal tract of rats in the high-fat diet group was reduced (P = 0.015), while the content of Verrucomicrobia increased (P = 0.035); In the comparison of genus content, Ruminococcus, Candida, Saccharibacteria genera incertae sedis, Enterobacter, Clostridium IV, Enterococcus, Enterorhabdus, Acetivibrio, Adlercreutzia, Lactococcus, etc., decreased significantly, while Akkermansia, Warthococcus, Staphylococcus, Butyricimonas, Clostridium XVIII, etc. increased significantly.

**Conclusion**: This study found that high-fat, high-calorie diet can increase the susceptibility of the intestine to carcinogenic factors. The reason may be that the high-fat diet causes the body to appear inflammatory states and microbial community imbalance, especially rumenococcus, candida, Saccharomyces, Enterobacter, Clostridium IV, Enterococcus, Enterobacter, Vibrioaceticus and other genus reduction are important links. Exploring ways to improve these floras is an important factor to improve the resistance of the intestinal tract to cancer-inducing agents.

## Introduction

High-fat and high-calorie diet is an important factor in the development of colorectal cancer, but the exact mechanism is still controversial. It is generally believed that high-fat and high-calorie diet causes hyperlipidemia and obesity, puts the body in a chronic inflammatory state, and may promote the intestine and thus stimulate the occurrence of tumors 1-2. Recent studies have shown that high-fat and high-calorie diets can cause changes in the abundance and types of microbial community, which may be an important cause of colorectal cancer 3-4. In our previous study,80% of the 159 colorectal patients have hyperlipidemia^4^.We also used tea extract to treat patients with hyperlipidemia, the serum lipid level ,insulin resistance and the inflammation factor such as TNF-α and IL-1 and found out that decreases^5^-^6^. However, we still did not know what is the exact the pathway of the high-fat and high-calorie diet, body inflammation state, changes in the intestinal flora and colorectal cancer. Is the colorectal cancer caused by changes in the intestinal flora itself? Or is it due to changes in the intestinal flora that the body's resistance to carcinogens changes, thereby making colorectal cancer more likely? There is no conclusion yet. This study investigated the relationship between changes in intestinal flora of rats under high-fat and high-calorie diet and tumorigenicity of carcinogens, and further explored the relationship between changes in intestinal flora under high-fat diet and carcinogenicity of carcinogens to provide new ideas for the prevention and treatment of colorectal cancer.

## Materials and methods

### 1. Feeding and grouping of experimental animals

40 SPF Wistar rats, male and female, 5-6 weeks old, weight 200g (animal certificate number: NO.11400700154060, experimental animal production license number: SCXK (Su) 2011-0003). Animal experiments were approved by the Animal Ethics Committee of the School of Medicine, Southeast University.

Animal diet was cobalt 60 radiation-sterilized pellets for rats and mice (Nanjing Jiangning Qinglongshan feed Company), high-fat feed (formulation: 20g lard, 5g cholesterol, 10g Tween 80, add water to 50ml).High-fat diet group: daily high-fat feeds were administered to the stomach. Gavage daily with warm saline in normal diet group.

Experimental animal grouping:

G1 group: normal diet group (control)

Group G2: normal diet + DMH

G3 group: high-fat diet group (control)

Group G4: high-fat diet + DMH

### 2. Animal treatment

At the 4th week of feeding, the mice were injected subcutaneously with dimethyl hydrazine (DMH) at 30 mg / kg twice a week. Dosing was continued for 8 weeks. The other groups were given weekly subcutaneous injections of the same amount of normal saline. Rats were weighed once a week to adjust the amount of DMH. At the 20th week of feeding, the rats were sacrificed.

### Detection of lipid metabolism and inflammatory factors

The blood was removed by eyeballs, and the plasma was separated by centrifugation, and the blood glucose and blood lipid metabolism indicators (total cholesterol, triolein, HDL, LDL) were detected. Inflammatory factors (IL-1, IL-6, COX-2) and cytokines (TNF-α, LEP) were detected by enzyme-linked immunosorbent assay.

### Fecal specimens and tissue specimens are retained

Before putting the animals to death, take 0.1 ~ 0.3g of fresh feces by massaging the abdomen, and immediately put them into liquid nitrogen and then store them in -80 ℃ refrigerator.

The rats were dissected, and the large intestine was cut freely and fully. The ascending colon, transverse colon, descending colon, and rectal intestinal mucosa were separated and stored in a -80 ° C refrigerator. The number and size of colon tumor in the rats were calculated and measured. Tumors or suspected tumors were completely removed and divided equally 2 parts, one part is stored in 10% formaldehyde and the other part is quickly frozen and transferred to -80 ℃ refrigerator for storage. The intestine is cut open longitudinally, flattened between two layers of filter paper and fixed at 10 % Neutral buffered formalin solution. Tumor size was measured and HE stained for pathological analysis.

### Research methods of coliform flora 8-9

Frozen fresh rat feces samples were quickly weighed and 0.1g samples were taken and centrifuged to extract DNA samples after rewarming.1ul MD microplate reader (MD company in the United States) was used to quantitatively detect the quality of DNA samples. After extraction, the DNA samples were expanded by 16sPCR (primer: 16s) -341F: CCTACGGGNGGCWGCAG; 16s- 341F: CCTACGGGNGGCWGCAG), the samples were sequenced according to the Illumina Miseq high-throughput sequencer usage guide. After running for 3-5 days, the original data was converted to Fastq format, and the data quality reached more than Q30 80%. In microbial diversity research, alpha diversity was measured by species richness (Richness, Chao, ACE) and diversity index (Shannon, Simpson); beta diversity analysis compares the similarity of species composition of samples, including cluster Class analysis and ranking analysis. The species abundance ratio chart counted each sample according to the boundaries, phylum, class, order, family, genus, and species level of the taxonomic unit of the species. Kruskal / wilcoxon rank sum test, similarity analysis (Anosim), Permanova substitution variance test, etc. were used to analyze the abundance and difference between species.

### Statistical methods

SPSS18.0 statistical software was used to analyze the data. The measurement data were all Mean ± SD. The comparison of multiple groups of data was performed by single factor analysis of variance. The comparison between groups was performed by LSD-t test. The repeated measurement data was analyzed by repeated measurement analysis of variance. When P <0.05, the difference was statistically significant.

## Results

### Tumor formation characteristics of rats in each group

**Figure 1:**
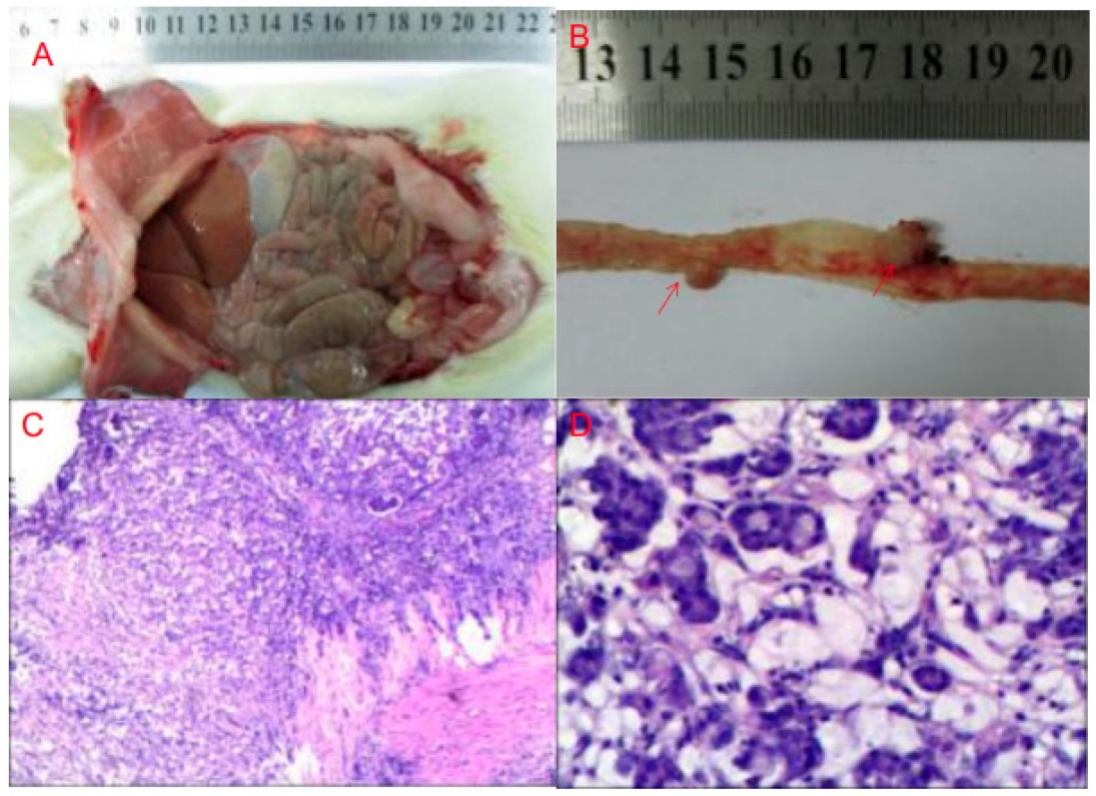
DMH-induced tumor formation in animal specimens and pathological HE staining results (A: gross finding B: cancer nodules red arrow showed C: cancer nodule ×200 D: cancer nodule ×400)

Rats gradually gained weight, but there was no significant difference between the 4 groups.There was no tumor formation in the normal diet group (G1) and high-fat diet group (G3). Four rats (40%) in the normal diet + DMH group (G2) formed 4 nodules, and the tumor weight was 0.06 ± 0.05g. Seven rats (70%) from fatty diet + DMH group (G4) formed 8 cancerous nodules with a tumor weight of 0.05 ± 0.03g. Compared with the normal diet cancer-inducing group, the high-fat diet cancer-inducing group had increased intestinal tumor formation (70% VS 40%) (Figure 1).

### Effects of high-fat diet on metabolism and inflammatory factors

Animals in the high-fat diet group increased cholesterol and TNF-α, while IL-1, IL-6, and LEP decreased. The difference was statistically significant. In the DMH-inducing group, only the difference in cholesterol was statistically significant (Table 1).

### Effect of high-fat diet on gut flora

Compared with the normal diet group (G1) and the high-fat diet group (G3), the intestinal bacterial abundance of rats was not significantly different, but the intestinal flora structure was significantly different (ANOSIM R = 0.2884, P = 0.011; PERMANOVA F = 3.750, P = 0.001).

**Figure 2:**
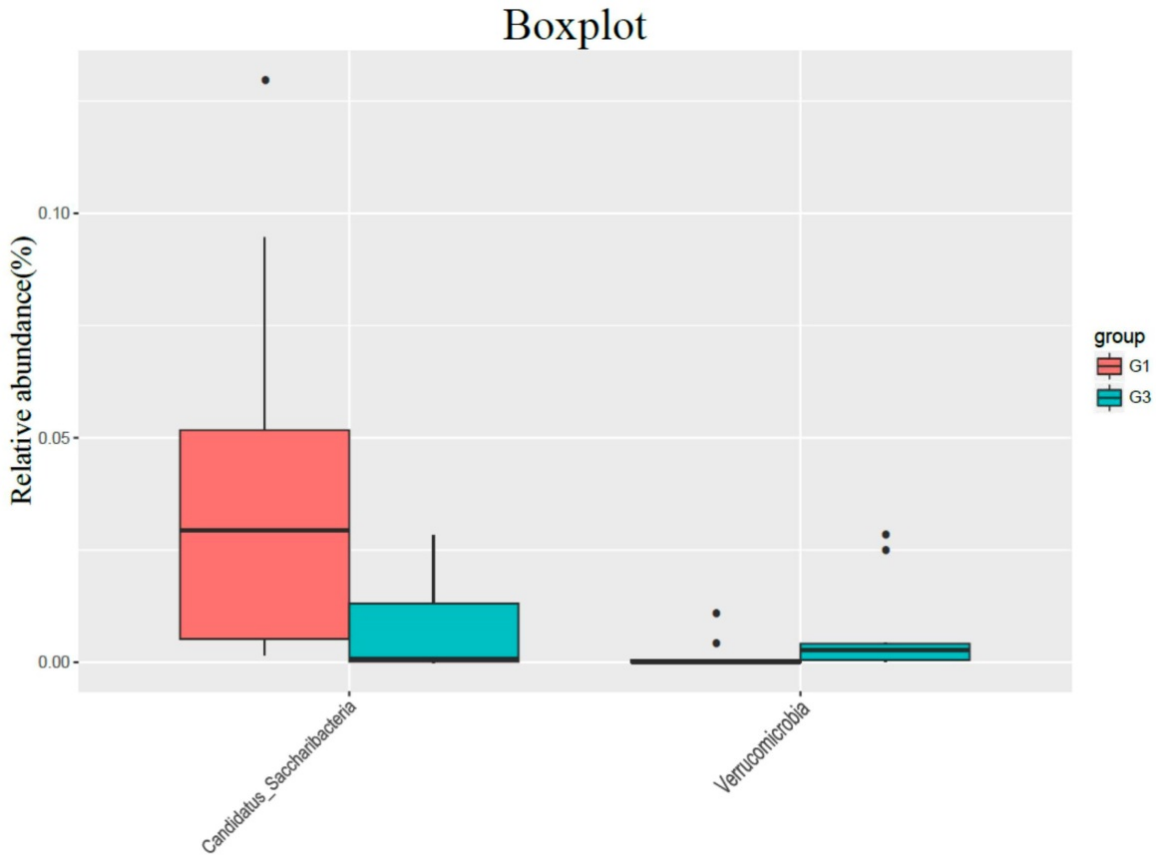
The influence of normal diet group (G1) and high-fat diet group (G3) on the flora

In the comparison of the phylum, the content of Candidatus Saccharibacteria in the high-fat diet group (G3) was significantly lower than that in the normal diet group (G1) (P = 0.015), and the content of Verrucomicrobia was significantly higher than that in G1 group (P = 0.035) (Figure 2).

In the comparison of bacterial species (genus) content, the rats in the high-fat diet group (G3) had significantly less gut bacterial species than the normal diet group (G1): Ruminococcus, Candida, Saccharibacteria genera incertae sedis, Enterobacter, Clostridium IV, Enterococcus, Enterorhabdus, Acetivibrio, Adlercreutzia, Lactococcus, Streptococcaceae, Clostridiales_Incertae_Sedis_XI, Gallicola, Coriobacteriales, Vagococcus, Pseudoflavonifractor, Slackia, Erysipelotrichaceae incertae sedis, Gallicola, Helicobacter, Unclassified Aerococcaceae, Unclassified Bdellovibrionales, Unclassified Clostridiales, Unclassified Ruminococcaceae.

**Figure 3:**
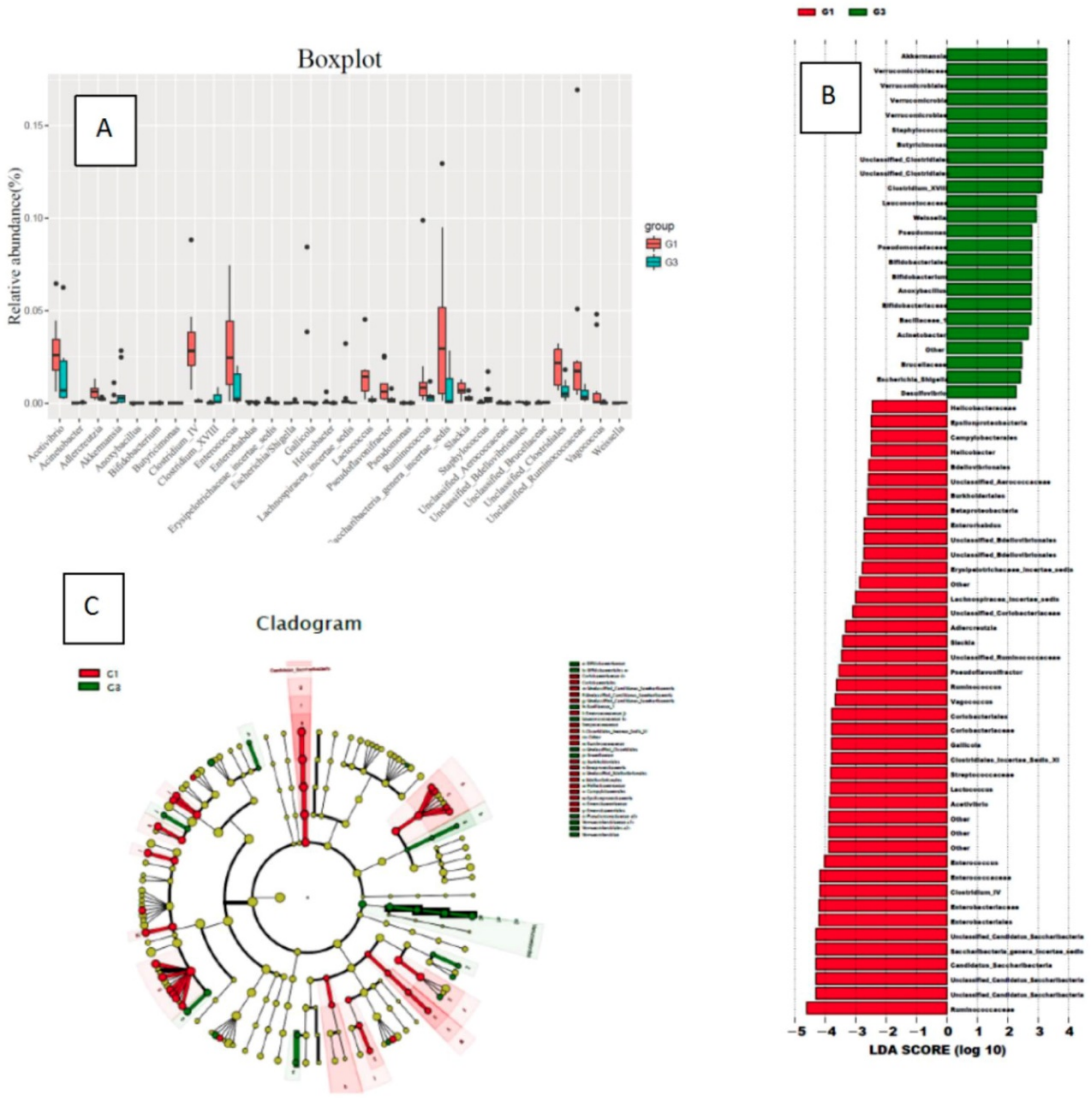
Changes in the species content of the bacterial flora in the normal diet group (G1) and the high-fat diet group (G3) (A: a comparison chart of the changes of each genus; B: an LDA chart of the changes of each genus; C: changes of each genus evolutionary branch diagram)

In the comparison of bacterial species (genus) content, the high-fat diet group (G3) rats had significantly increased gut bacterial species compared to the normal diet group (G1): Akkermansia, Verrucococcus, Staphylococcus, Butyricimonas, Clostridium XVIII, Leuconostocaceae, Weissella, Pseudomonas, Bifidobacterium, Anoxybacillus, Acinetobacter, Anoxybacillus, Escherichia/Shigella, Unclassified Brucellaceae. Compared with the G1 group, the content increased significantly (Figure 3).

### Effect of carcinogen DMH on the gut flora of animals in normal diet group and high-fat diet group

**Figure 4:**
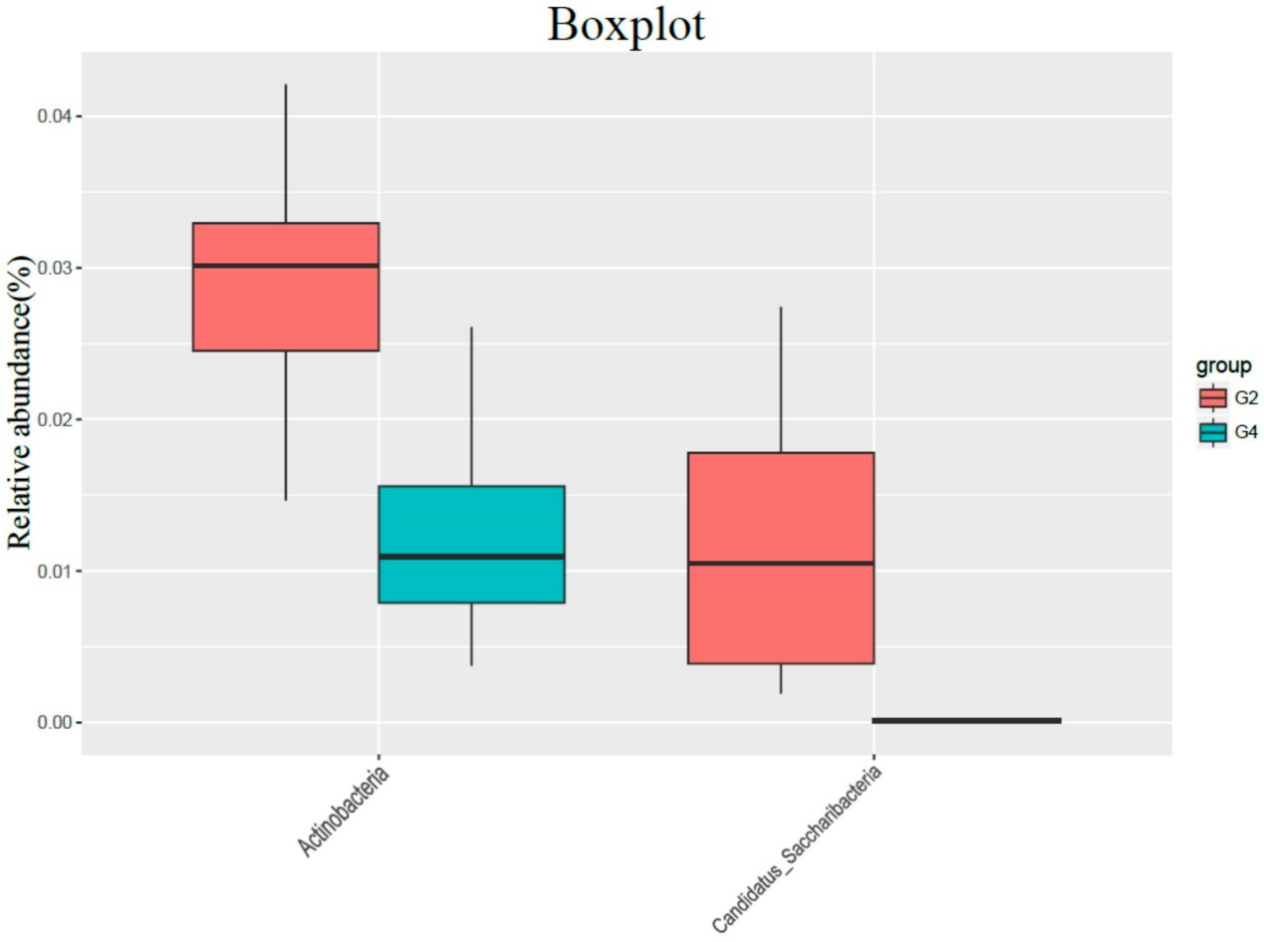
The influence of normal diet group (G2) and high-fat diet group (G4) on the flora

Compared with G4 group, G2 group had no significant difference in gut bacterial abundance, but the intestinal flora structure changed significantly (ANOSIM R = 0.292, P = 0.001; PERMANOVA F = 3.697, P = 0.001). In the comparison of bacteria categories, the contents of Actinobacteria and Candida in the G4 group were significantly lower than those in the G2 group (Figure 4).

In the comparison of bacterial species content, the bacteria that were significantly reduced in the G4 group compared to the G2 group were: Ruminococcus, Vibrio acetate, Adlercreutzia, Clostridium IV, Enterorhabdus, Erysipelotrichaceae incertae sedis, Facklamia, Leafworm, Gordonibacter, Helicobacter, Lachnospiracea incertae sedis, Lactococcus, Oligobacterium, Pseudoflavonifractor, Saccharibacteria genera incertae sedis, Saccharibacteria genera incertae sedis, Slackia, Nonclass, Unclassified Bacteroides, Unclassified Bdellovibrionales, Unclassified Clostridium, Unclassified Coriobacteriaceae and unclassified rumenaceae.

**Figure 5:**
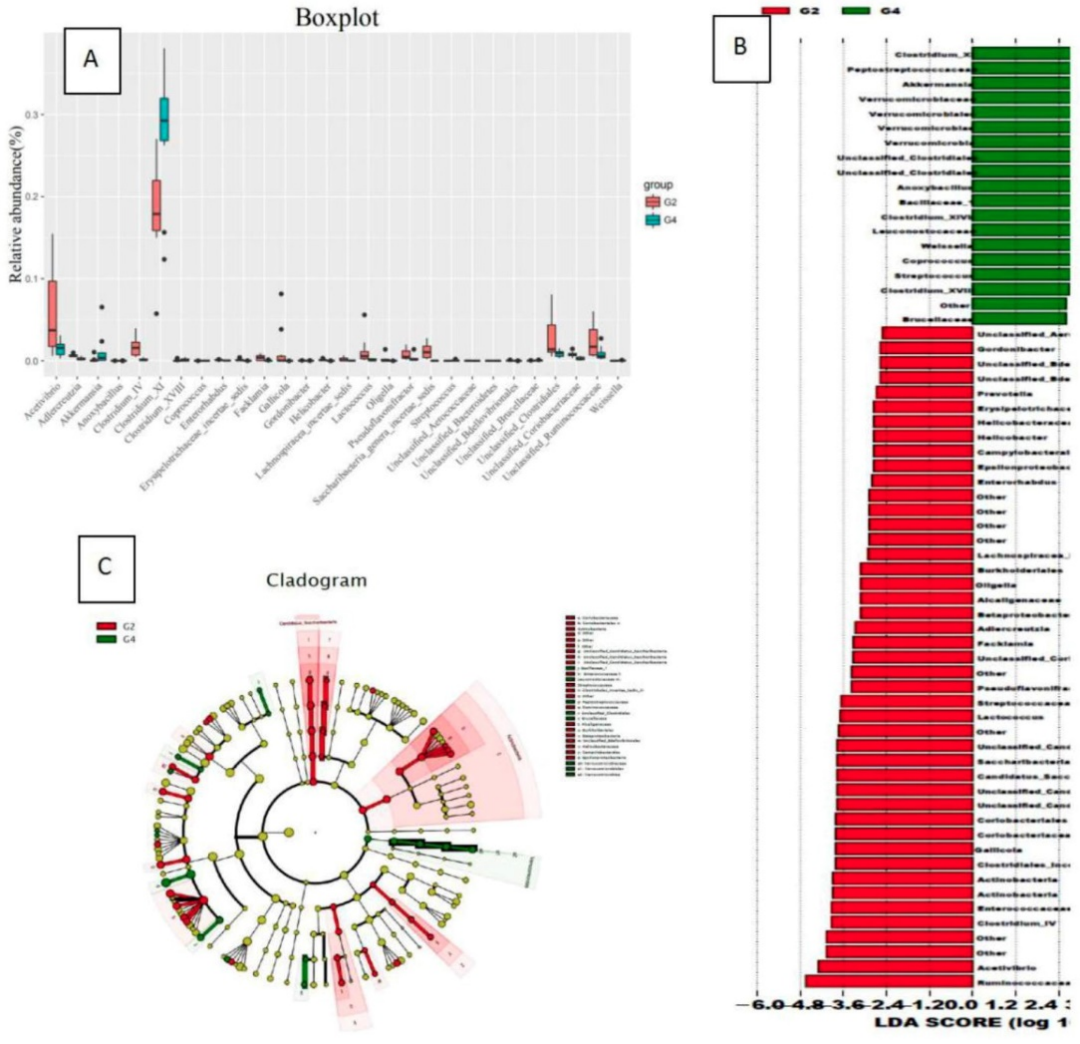
The effect of normal diet + DMH group (G2) and high-fat diet group + DMH group (G4) on the types of flora (Evolving branch diagram of changes in each genus)

The bacteria that significantly increased in the G4 group compared to the G2 group were: Clostridium XI, Streptococcus pepticus, Ackermania, Micrococcus versicolor, Clostridium XVIII, Coprococcus, Streptococcus (Figure 5).

## Discussion

High-fat and high-calorie diet plays an important role in the development of colorectal cancer, but there is no clear evidence on how high-fat and high-calorie diet can cause colorectal cancer 10-12. High-fat and high-calorie diets cause hyperlipidemia in the body, which in turn causes the body to be in a chronic inflammatory state. But can high-fat and high-calorie diets alone cause the occurrence of colorectal tumors?

From the research in this group, compared with the normal diet group, the high-fat and high-calorie diet group had hyperlipidemia and inflammatory factors such as TNF-α increased.But after 20 weeks of observation, the animals did not develop colorectal cancer,which suggested that only high-fat, high-calorie diet will not cause colorectal tumor for a short period of time. In the literature, there have been few reports of the use of high-fat diet alone to induce colorectal tumors 13-14. Current research suggests that a high-fat, high-calorie diet can cause the body to be in an inflammatory state, which may cause the body to be susceptible to cancer 15-17. Tuominen et al. 18 studied AOM-induced colorectal cancer in animals using high-fat and high-calorie diets. If the high-fat and high-calorie diet was switched to a normal diet at the beginning of the cancer-inducing experiment, the risk of colorectal cancer in animals would not be significantly reduced. Therefore, the author believed that the high-fat and high-calorie diet played a role in colorectal tumor which may be an early stage affair. Xiu et al. 3 also considered that a high-fat, high-calorie diet can promote tumor growth in tumor-bearing animals.

In general, hyperlipidemia caused by a high-fat, high-calorie diet may put the body in a chronic inflammatory state, increases the body's sensitivity to carcinogenic factors, and makes the body more prone to tumors 15-17. In our study, the incidence of colorectal cancer in the normal diet group with DMH was 40% (4/10), while the incidence of colorectal cancer in the high-fat diet group with DMH was 70% (7/10), and the tumor formation rate increased significantly. It implied that the high-fat and high-calorie diet causes the body to be in a chronic inflammatory state and may increase the sensitivity to carcinogens.

Modern research has shown that chronic inflammatory station leading to imbalance of the coliform flora may be an important cause of susceptibility to colorectal cancer 17-18. In this group of studies, although the bacterial abundance of animals in the high-fat diet group did not change significantly, the types and distribution of bacteria changed significantly. In the comparison of gut microbiome, the content of candida in the intestine of rats in the high-fat diet group was significantly reduced compared with the normal diet group, while the content of Verrucomicrobia was significantly increased compared with the normal diet group. Verrucomicrobia is a kind of bacteria enriched in the intestinal mucosa, which will decrease in high-fat diets, obesity and other diseases. This study is similar to reports in the literature 19.

At the species level, rumenococcus, candida, saccharin, enterobacteria, clostridium IV, enterococcus, enterobacteria, vibrioaceticus, etc. were reduced in animals of the high-fat and high-calorie diet group. Most of these bacteria are already reported probiotics. The reduction of these bacteria may cause intestinal microenvironment disorders, and the intestinal mucosa barrier to resist external invasion will decrease, leading to increased susceptibility to carcinogenic factors 20-21. After using DMH, the proportion of rumenococcus, Candida, Clostridium IV, and Enterobacter bacteria in the high-fat diet group decreased further, further indicating that the decrease in the proportion of these bacteria is an important cause of the increased intestinal susceptibility to carcinogens the reason.

In the high-fat and high-calorie diet group, bacterial genera such as Akkermansia, Warthococcus, and Staphylococcus increased. The increase in the proportion of these bacterial genera may be related to the decrease in the proportion of other bacterial genera. In this group of results, the content of Akkermansia increased in high-fat and high-calorie diets 22-23, and the literature reported that in high-fat and high-calorie diets and metabolic diseases, proportion of Akkermansia may decrease. The results were different from those reported in the literature. The reason was not clear. It may be related to the significant decrease in the proportion of other bacteria and lead to increase Akkermansia relatively in this group of studies. Further research will be conducted to conform our outcome. After the use of DMH to induce cancer, bacteria such as Clostridium XI, Streptococcus digesta, Ackermania, Clostridium XVIII increased, suggesting that these bacteria play a harmful role in the intestinal, which may be the reason of the intestinal tract to carcinogens.

Due to the complexity of the intestinal flora, a series of bacteria rise and fall after a high-fat, high-calorie diet but how these bacteria work and what role they play is currently unclear. From the perspective of clinically regulating the intestinal microecology, selective fecal transplantation can be used to supplement the decreased bacteria, but there is currently no better way to remove the increased bacteria 24.Therefore, it is significant to explore the decreased beneficial bacteria and find ways to improve these.

In summary, this study shows that high-fat and high-calorie diets can increase the susceptibility of the intestines to carcinogenic factors, which may be due to high-fat diets leading to inflammatory states and intestinal flora imbalances, especially rumenococcus, candida, Saccharomyces, Enterobacter, Clostridium IV, Enterococcus, Enterobacter, Vibrioaceticus and other genus reduction are important links. Exploring ways to improve these flora is an important factor to improve the resistance of the intestinal tract to cancer-inducing agents.

## Figures and Tables

**Table 1:** Comparison between lipid metabolism and serum inflammation corresponding groups (n = 40)

	G1	G3	P(G1vs G3)	G2	G4	P(G2vsG4)
Glucose	9.57±2.66	6.89±0.64	0.619	7.81±1.19	7.40±1.72	0.909
Triglyceride	0.93±0.30	0.58±0.09	0.366	0.58±0.26	0.70±0.23	0.697
cholesterol	6.02±1.04	7.38±1.61	0.002	5.85±1.31	8.19±2.65	0.031
LDL	6.57±2.10	5.32±3.24	0.550	4.75±1.75	6.70±2.86	0.399
HDL	2.42±1.02	2.07±1.14	0.724	1.74±0.40	2.68±1.45	0.105
IL-1	35.61±13.84	24.83±12.89	0.001	21.68±11.71	21.49±10.84	0.971
IL-6	9.55±1.77	7.52±1.57	0.001	7.66±1.66	7.03±1.25	0.385
LEP	1.71±0.29	1.36±0.55	0.013	1.38±0.22	1.34±0.37	0.885
TNF-α	28.00±23.02	45.73±13.23	0.018	43.23±14.08	43.70±10.40	0.949

G1, normal diet group (blank control); G2, normal diet + DMH; G3, high fat diet group (blank control); G4, high fat diet + DMH

## Abbreviations

HFD: high-fat diet
DMH: dimethyl hydrazine
TNF: tumor necrosis factor
LEP: leptin
IL: interleukin
HDL: high density lipoprotein
LDL: low density lipoprotein
COX: cyclooxygenase
